# Causal associations of air pollutants with chest and gingival pain: Genetic insight from Mendelian randomization study

**DOI:** 10.1097/MD.0000000000044258

**Published:** 2025-09-05

**Authors:** Xian-Pei Xiao, Rui Liang, Yin-Fei Luo, Xiu-Ming Li, Yao Li, Yuan Liu, Xiao-Li Zhou, Zhi-Heng Li

**Affiliations:** a Department of Critical Care Medicine, Luojiang District People’s Hospital of Deyang City, Deyang, Sichuan, China; b Department of Radiology, Affiliated Hospital of North Sichuan Medical College, Nanchong, Sichuan, China; c Department of Orthopedics, Luojiang District People’s Hospital of Deyang City, Deyang, Sichuan, China; d Department of Neurosurgery, Luojiang District People’s Hospital of Deyang City, Deyang, Sichuan, China; e Department of Basic Medicine and Law, School of North Sichuan Medical College, Nanchong, Sichuan, China.

**Keywords:** air pollutants, chest pain, gingival pain, Mendelian randomization

## Abstract

Epidemiological studies have already established associations between air pollutants and adverse health outcomes, but the causal associations between air pollutants and chest pain (CP) and gingival pain (GP) remain unclear. This study aimed to explore the potential causal effects of air pollutants on CP and GP. Utilizing genome-wide association study summary statistics from European-ancestry populations, we conducted bidirectional two-sample Mendelian randomization (MR) analyses. Causal estimates were primarily derived through inverse-variance weighted regression, weighted median, MR-Egger, and weighted mode methods to assess pleiotropy and robustness. Multivariable MR (MVMR) further adjusted for smoking and alcohol consumption covariates. MR analyses demonstrated significant causal effects of particulate matter (PM) 2.5 exposure on both CP (OR = 1.060, 95% CI: 1.036–1.085, *P *= 5.51 × 10^−07^) and GP (OR = 1.031, 95% CI: 1.008–1.056, *P *= .008). Further MVMR analysis supported that the causal associations of PM_2.5_ exposure with CP and GP persisted after controlling for smoking and alcohol consumption. No significant causal effects were observed for PM10, PM2.5-10, or nitrogen oxides exposure. Our findings provide novel genetic evidence that long-term PM₂.₅ exposure independently increases risks of CP and GP, underscoring the need for targeted air quality interventions and public health strategies to mitigate particulate matter-related disease burden.

## 1. Introduction

Chest pain (CP) represents a prevalent clinical manifestation encompassing multiple thoracic and abdominal organ systems, accounting for approximately 60,000 annual emergency department presentations in the United States.^[1–6]^ While the precise etiology of CP is undetermined, existing literature predominantly associates CP with cardiovascular issues. Cardiovascular disorders, including angina pectoris, myocardial infarction, and heart failure, are diagnosed in over 50% of CP patients presenting to emergency departments.^[7,8]^ Additionally, CP is linked to pulmonary embolism, musculoskeletal disorders, and gastrointestinal issues, which are common diagnoses in outpatient settings.

Gingival pain (GP) is characterized by redness, swelling, and stinging in the gums, primarily resulting from inflammation or trauma. Similar to CP, GP exhibits complex etiopathogenesis, with dental caries, periodontitis, and gingivitis serving as primary contributing factors.^[9]^ Some evidence suggests a potential link between CP and GP. A cohort study found a positive association between periodontitis and the risk of atrial fibrillation, while a meta-analysis indicated that periodontitis patients have a higher risk of developing atherosclerotic cardiovascular disease.^[10]^ Environmental factors are known to cause various diseases. Thus, we focused on exploring the effects of air pollutants on CP and GP.

Particulate matter (PM) and gases, primarily produced by fuel combustion and transportation emissions, constitute the complex class of mixtures known as air pollutants.^[11]^ Air pollutants are a significant environmental risk factor for noncommunicable diseases, second only to tobacco smoking, and have been linked to the onset and progression of numerous diseases.^[12]^ A study conducted in Taiwan, China, revealed that elevated levels of PM2.5 exposure heightened the risk of hospital admissions for CP, particularly during warmer seasons.^[13]^ Another study reported that the incidence of CP increased by 38% for every 5.12 µg/m^3^ increase in PM_2.5_ concentration.^[14]^ Although there is no direct evidence linking air pollutants to GP, cigarette smoke, similar in composition to air pollutants, can harm gum tissue and cause gingival hyperpigmentation.^[14]^ Cellular experiments have confirmed this, showing that cigarette smoke induces apoptosis in gingival epithelial cells and disrupts cell repair.^[15]^ While strong evidence linking air pollution to CP and GP is lacking, current literature suggests potential adverse effects of air pollutant exposure on the pathogenesis of these diseases.^[16]^

Mendelian randomization (MR) analysis is a powerful epidemiological approach that uses genetic variants as instrumental variables (IVs) to assess causal relationships between exposures and outcomes.^[17]^ This method addresses key limitations of traditional observational studies by minimizing confounding bias and reverse causality through the inherent randomness of genetic inheritance. While accumulating evidence has linked air pollution to various inflammatory conditions, the specific causal effects of PM components on CP and GP remain underexplored. Our study aimed to systematically evaluate the causal effects of 4 air pollutants (PM2.5, PM10, PM2.5-10, and nitrogen oxides [NOx]), on the risks of CP and GP, to raise public awareness of the hazards of air pollutants.

## 2. Methods

### 2.1. Source of data

**Figure 1.**
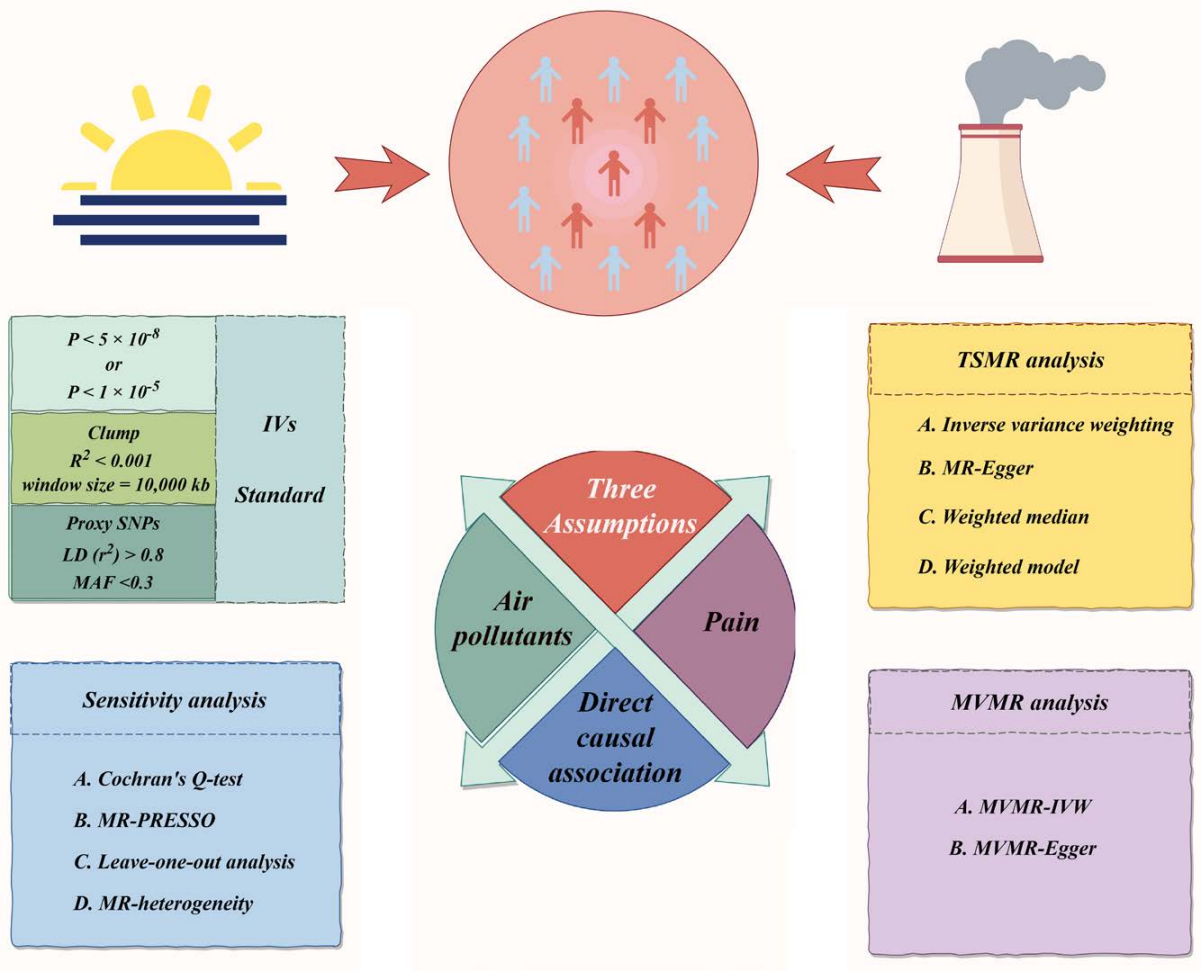
The study design of causal inference between air pollutants and the risk of CP and GP. CP = chest pain, GP = gingival pain, IVs = instrumental variables, IVW = inverse variance weighting, LD = linkage disequilibrium, MAF = minor allele frequency, MR = Mendelian randomization, MVMR = multivariable Mendelian randomization, PM = particulate matter, SNPs = single nucleotide polymorphisms, TSMR = two sample MR.

Exposure datasets for 4 air pollutants, including PM2.5, PM10, PM2.5-10, and NO_X_ were obtained from the IEU Open Genome-wide Association Study (GWAS) database (https://gwas.mrcieu.ac.uk/datasets). The datasets for PM2.5, PM10, and PM2.5-10 included 423,796 participants, while the NO_X_ dataset had 456,380 individuals, all of European descent. These GWAS datasets, part of the European Study of Cohorts for Air Pollution Effects, which employed a land use regression modeling to estimate residential exposure levels based on participants’ geographical coordinates.^[18]^ Figure 1 illustrates the potential mechanisms of air pollutants and their impact on CP and GP, along with the MR analysis process.

Genetic outcome data for CP and GP were derived from the UK Biobank datasets, a large prospective study with over 500,000 European participants. The GWAS data for CP includes 18,203 cases and 444,807 controls, while the data for GP comprises 13,314 cases and 447,799 controls. To mitigate population stratification bias, both exposure and outcome GWAS data were sourced exclusively from studies involving individuals of European ancestry (Table S1, Supplemental Digital Content, https://links.lww.com/MD/P868).

### 2.2. Study design and IVs selection

To ensure the authenticity of causal estimates, IVs must satisfy 3 core assumptions: strong association with the exposure of interest (relevance assumption); independence from measured or unmeasured confounders affecting the exposure-outcome relationship (dependence assumption); and exclusivity of effect on the outcome through the exposure pathway without alternative biological mechanisms (exclusion restriction).^[19]^ We implemented a multi-step protocol to rigorously select IVs meeting these criteria. First, we set a significance threshold of *P* < 5 × 10^−08^ for the selection of exposure-related single nucleotide polymorphisms (SNPs). However, as there were no eligible SNPs identified for PM10 and PM2.5-10, the broadened threshold of *P* < 1 × 10^−05^ was implemented. Second, we applied a stringent standard (*R*^2^ < 0.001 within 10,000 kb windows) to remove any linkage disequilibrium among the selected SNPs. Third, missing SNPs in outcome datasets were replaced with high-quality proxy variants with linkage disequilibrium *r*^2^ values over than 0.8 and a minor allele frequency threshold for aligning palindromes at 0.3. Finally, to avoid the effects of weak IVs, we calculated *F*-statistics of each IVs was calculated as follows^[20]^:


$$\begin{array}{l} F=R^{\text{2}}\times (N-2)/(1-R^{\text{2}}), \end{array}$$

$$\begin{array}{l} \begin{array}{lll} R^{2}= & 2\times beta^{2}\times eaf\times (1\text{-}eaf)/[2\times beta^{2}\; & \times eaf\times (1\text{-}eaf)+2\times se^{2}\times N\times eaf\times (1\text{-}eaf)],\; \end{array} \end{array}$$

only those IVs with the *F*-value over than 10 were selected in MR analysis.

### 2.3. Ethical approval

All data used in this study were publicly available GWAS abstracted data; therefore, no additional informed consent or ethical approval was required.

### 2.4. Statistical analysis

#### 2.4.1. MR analysis

In this study, we utilized several complementary MR methods to infer the causal relationships of air pollutants with CP and GP, including inverse-variance weighted (IVW), weighted median (WM), MR-Egger and weighted mode method. The IVW method was prioritized as the primary analytical framework due to its optimal statistical power and reliability in scenarios without significant horizontal pleiotropy or heterogeneity.^[21]^ The MR-Egger method provided pleiotropy detection through its intercept test while maintaining consistent causal estimates even with invalid IVs, albeit with reduced precision.^[22]^ The MR-Egger method provided pleiotropy detection through its intercept test while maintaining consistent causal estimates even with invalid IVs, albeit with reduced precision.^[23]^ The weighted mode method served as supplementary validation with its bias-resistant design, though with relatively lower statistical power.^[24]^ A hierarchical analytical strategy was adopted to ensure result robustness. When no pleiotropy was detected, IVW estimates were considered definitive. In cases of suspected pleiotropic effects, WM results superseded IVW findings due to their inherent resistance to invalid instruments. Final causal determinations required concurrent statistical significance (*P* < .05) from both IVW and WM analyses.

#### 2.4.2. Sensitivity analysis

To evaluate heterogeneity across IVs, we performed Cochran Q-test with a threshold of *P* < .05 indicating significant heterogeneity.^[25]^ Directional pleiotropy was systematically examined through 2 complementary approaches. First, the intercept term from MR-Egger regression was analyzed, with a value approximating zero (*P* > .05) indicating absence of detectable pleiotropic effects.^[26]^ Second, the MR pleiotropy residual sum and outlier (MR-PRESSO) framework was implemented to simultaneously detect outlier variants and provide bias-corrected estimates through distortion testing and outlier removal.^[27]^ Furthermore, leave-one-out sensitivity analyses were conducted to verify result consistency by iteratively excluding individual genetic variants. Our study ensures the reliability of causal inference through multiple sensitivity analysis of heterogeneity assessment, pleiotropy detection and robustness verification.

#### 2.4.3. Multivariate MR (MVMR) analysis

To account for potential confounding effects from coexisting risk factors and interactions between air pollutants, we performed MVMR analysis. This approach allowed us to examine the direct causal associations of individual air pollutants with the risks of CP and GP while adjusting for specific PM fractions and significant behavioral covariates (smoking and alcohol consumption).^[28]^ Given the multiplicity of statistical comparisons, we implemented the false discovery rate method to correct *P*-values, considering an false discovery rate-adjusted *P*-value < .05 as statistically significant. The “TwoSampleMR” package (version 0.5.6, University of Bristol, Bristol, United Kingdom) was employed for conducting basic MR analyses. The “MendelianRandomization” package (version 0.5.1, University of Cambridge, Cambridge) was utilized to validate the results of these analyses. The “MR-PRESSO” package (version 1.0, McGill University, Montreal) was applied to detect and correct any potential biases. The “ggplot2” package (version 3.4.0, Rice University, Houston) was used for visualizing the results. All analyses were conducted within the R software environment (version 4.2.2, R Foundation for Statistical Computing, Vienna, Austria).

## 3. Results

A total of 8 independent SNPs was initially selected as IVs for PM2.5 and NO_X_ based on the strict threshold of *P* < 5 × 10^−08^, respectively. However, as there were no independent SNPs that reached a genome-wide association (*P* < 5 × 10^−08^) for PM10 and PM2.5-10, we broaden the threshold to *P* < 1 × 10^−05^ for IVs selection, and observed 58 PM10-associated SNPs and 41 PM2.5-10-associated SNPs, respectively. A single SNP (rs1039952) in PM10 was excluded due to an echo structure. After matching with the outcome datasets of CP, a number of 7 PM2.5-associated SNPs, 42 PM10-associated SNPs, 34 PM2.5-10-associated SNPs and 8 NO_X_-associated SNPs were incorporated into MR analysis. Along with the associated SNPs in the CP dataset, 7 PM2.5-associated SNPs, 39 PM10-associated SNPs, 31 PM2.5-10-associated SNPs, and 8 NO_X_-associated SNPs were correctly matched in the dataset of GP. None of the missing SNPs were found to be replaced by suitable proxy SNPs. All included IVs had a *F*-value >10, suggesting that the causal estimates might not prone to the influences of weak IVs. Tables S2 and S3, Supplemental Digital Content, https://links.lww.com/MD/P868 provided summaries of the detailed information for the included IVs.

### 3.1. Causal associations between air pollutants and CP

**Figure 2.**
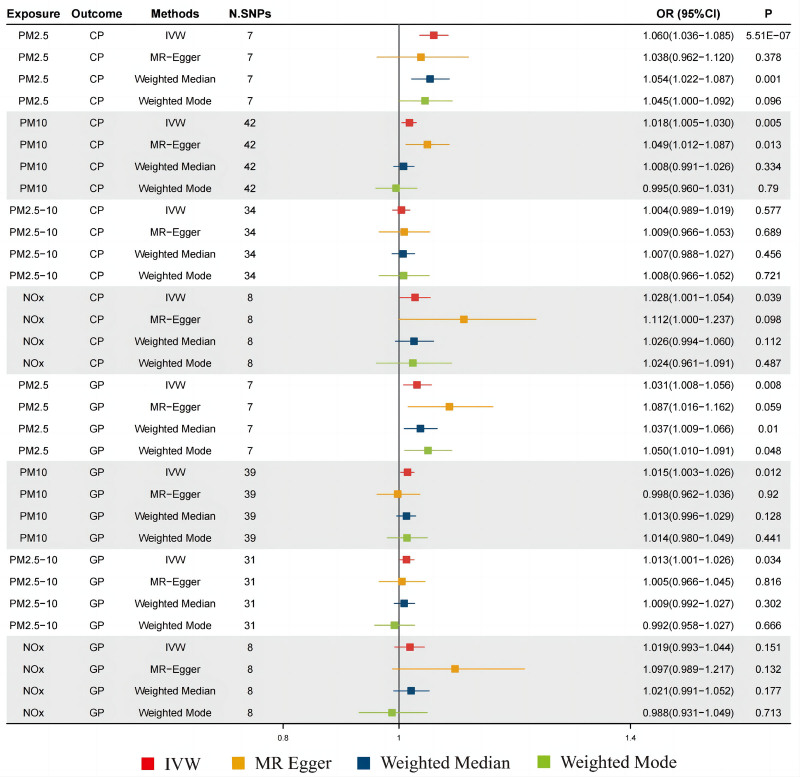
Estimated causal effects between air pollutants and CP, GP using different MR methods. CP = chest pain, GP = gingival pain, IVW = inverse variance weighted, MR = Mendelian randomization, N. SNPs = number of SNPs used in MR, NO_X_ = nitrogen oxides, OR = odds ratio, PM = particulate matter.

**Figure 3.**
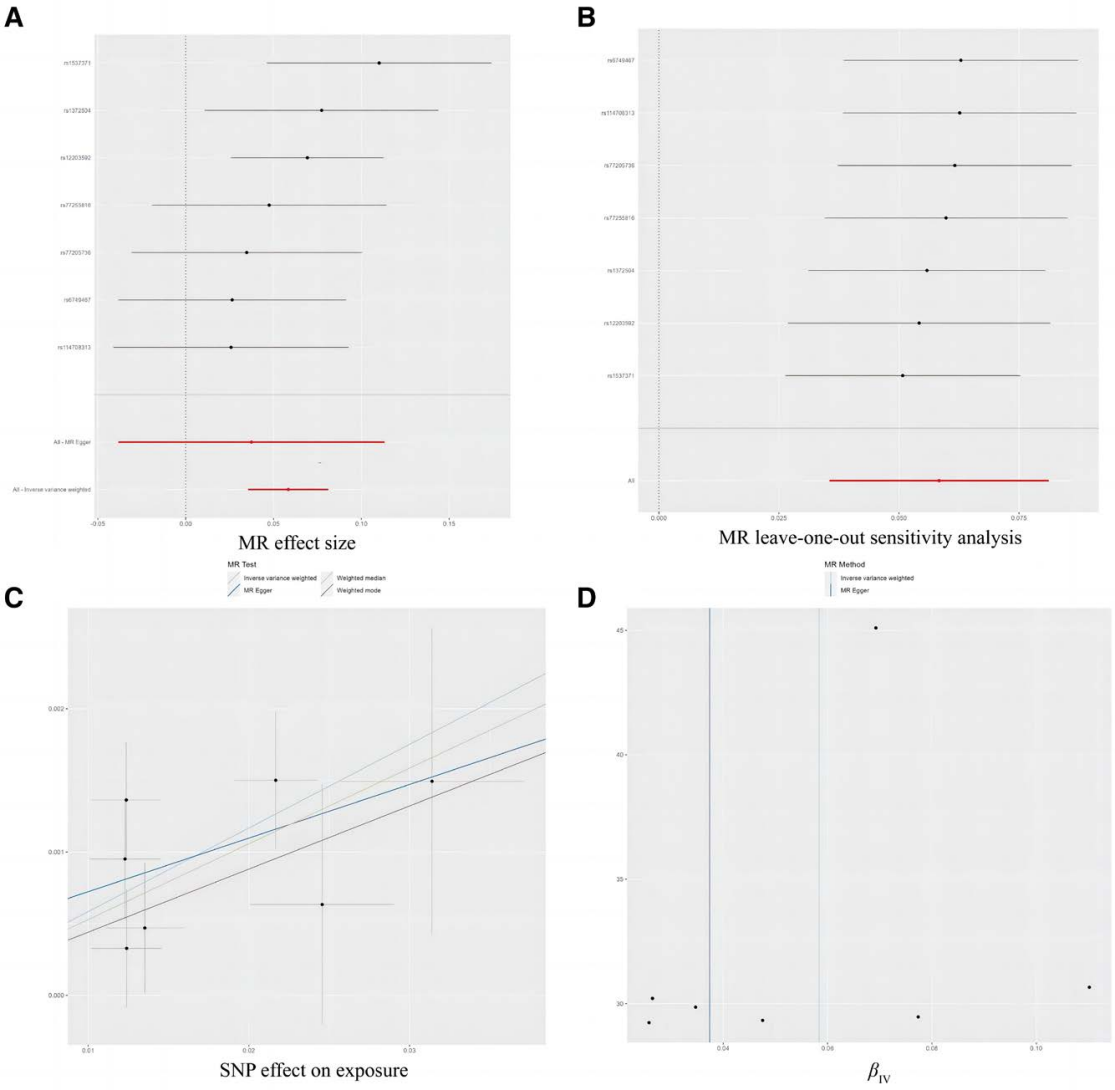
Forest plot (A), sensitivity analysis (B), scatter plot (C), and funnel plot (D) of the causal effect of PM2.5 on chest pain risk. CP = chest pain, GP = gingival pain, MR = Mendelian randomization, PM2.5: particulate matter 2.5, SNPs = single nucleotide polymorphisms.

Exposure to PM_2.5_ was causally linked to a higher chance of developing CP (odds ratio [OR]_IVW_ = 1.060, 95% CI = confidence interval [CI]: 1.036–1.085, *P *= 5.51 × 10^−07^; OR_WM_ = 1.054, 95% CI: 1.022–1.087, *P *= .001; Figs. 2 and 3), this result remained consistent after correcting for *P*-value due to multiple tests (Table 1). Sensitivity analysis with leave-one-out method implied that the observed causal association was not influenced by any single SNP (Fig. 3). In addition, the results based on Cochran *Q*-test, MR-Egger regression and MR-PRESSO tests indicated that the observed causal association was robust, and there were no effect of horizontal pleiotropy and strong heterogeneity (Table 2). In addition, the IVW method likewise found the causality of exposures to PM10 and NO_X_ with the risk of CP (PM10: OR_IVW_ = 1.018, 95% CI: 1.005–1.030, *P *= .005; NO_X_: OR_IVW_ = 1.028, 95% CI: 1.001–1.054, *P *= .039), but this result was not supported by WM method. Finally, no causal association between PM2.5-10 and CP was observed. The depicted forest plots, sensitivity analysis, scatter plots and funnel plots regarding the causal relationship between the 4 pollutants and CP were shown in Figure 3 and Figures S1–S3, Supplemental Digital Content, https://links.lww.com/MD/P867, respectively.

**Table 1:** Causal effects of air pollutants on CP and GP with different MR methods.

Exposure	Outcome	N. SNPs	Methods	OR	(95% CI)	*P* value	FDR-*P*
PM_2.5_	CP	7	IVW	1.060	(1.036–1.085)	5.51E−07	1.10E−06
		7	MR Egger	1.038	(0.962–1.120)	.378	.378
		7	WM	1.054	(1.022–1.087)	.001	.002
		7	Weighted mode	1.045	(1.000–1.092)	.096	.096
PM_10_	CP	42	IVW	1.018	(1.005–1.030)	.005	.010
		42	MR Egger	1.049	(1.012–1.087)	.013	.026
		42	WM	1.008	(0.991–1.026)	.334	.334
		42	Weighted mode	0.995	(0.960–1.031)	.790	.790
PM_2.5-10_	CP	34	IVW	1.004	(0.989–1.019)	.577	.577
		34	MR Egger	1.009	(0.966–1.053)	.689	.816
		34	WM	1.007	(0.988–1.027)	.456	.456
		34	Weighted mode	1.008	(0.966–1.052)	.721	.721
NO_X_	CP	8	IVW	1.028	(1.001–1.054)	.039	.078
		8	MR Egger	1.112	(1.000–1.237)	.098	.132
		8	WM	1.026	(0.994–1.060)	.112	.177
		8	Weighted mode	1.024	(0.961–1.091)	.487	.713
PM_2.5_	GP	7	IVW	1.031	(1.008–1.056)	.008	.008
		7	MR Egger	1.087	(1.016–1.162)	.059	.118
		7	WM	1.037	(1.009–1.066)	.010	.010
		7	Weighted mode	1.050	(1.010–1.091)	.048	.096
PM_10_	GP	39	IVW	1.015	(1.003–1.026)	.012	.012
		39	MR Egger	0.998	(0.962–1.036)	.920	.920
		39	WM	1.013	(0.996–1.029)	.128	.256
		39	Weighted mode	1.014	(0.980–1.049)	.441	.790
PM_2.5-10_	GP	31	IVW	1.013	(1.001–1.026)	.034	.068
		31	MR Egger	1.005	(0.966–1.045)	.816	.816
		31	WM	1.009	(0.992–1.027)	.302	.456
		31	Weighted mode	0.992	(0.958–1.027)	.666	.721
NO_X_	GP	8	IVW	1.019	(0.993–1.044)	.151	.151
		8	MR Egger	1.097	(0.989–1.217)	.132	.132
		8	WM	1.021	(0.991–1.052)	.177	.177
		8	Weighted mode	0.988	(0.931–1.049)	.713	.713

CI = confidence interval, CP = chest pain, FDR = false discovery rate, GP = gingival pain, IVW = inverse variance weighted, MR = Mendelian randomization, N. SNPs = number of SNPs used in MR, NO_X_ = nitrogen oxides, OR = odds ratio, PM = particulate matter, WM = weighted median.

**Table 2:** The results of the pleiotropy and sensitivity analysis.

Exposure	Outcome	N. SNPs	IVW			MR-Egger intercept			MR-PRESSO	
			OR (95% CI)	Q statistic	Q_pval	Intercept	SE	*P* value	RSSobs	*P* value
PM_2.5_	CP	7	1.060 (1.036–1.085)	5.512	0.480	0.0003	0.0006	.594	7.325	.570
	GP	7	1.031 (1.008–1.056)	8.200	0.224	−0.0008	0.0005	.170	11.526	.245
PM_10_	CP	42	1.018 (1.005–1.030)	39.177	0.552	−0.0005	0.0003	.087	41.245	.602
	GP	39	1.015 (1.003–1.026)	40.978	0.341	0.0002	0.0003	.369	46.180	.286
PM_2.5-10_	CP	34	1.004 (0.989–1.019)	41.281	0.153	−0.00007	0.0003	.824	43.886	.155
	GP	31	1.013 (1.001–1.026)	22.471	0.836	0.0001	0.0003	.663	24.004	.824
NO_X_	CP	8	1.028 (1.001–1.054)	9.034	0.250	−0.001	0.0008	.185	12.952	.228
	GP	8	1.019 (0.993–1.044)	11.349	0.124	−0.001	0.0008	.201	15.799	.111

CI = confidence interval, CP = chest pain, GP = gingival pain, IVW = inverse variance weighted, MR = Mendelian randomization, MR-PRESSO = Mendelian randomized residuals and outliers, N. SNPs = number of SNPs used in MR, NO_X_ = nitrogen oxides, OR = odds ratio, PM = particulate matter.

### 3.2. Causal association between air pollutants and GP

**Figure 4.**
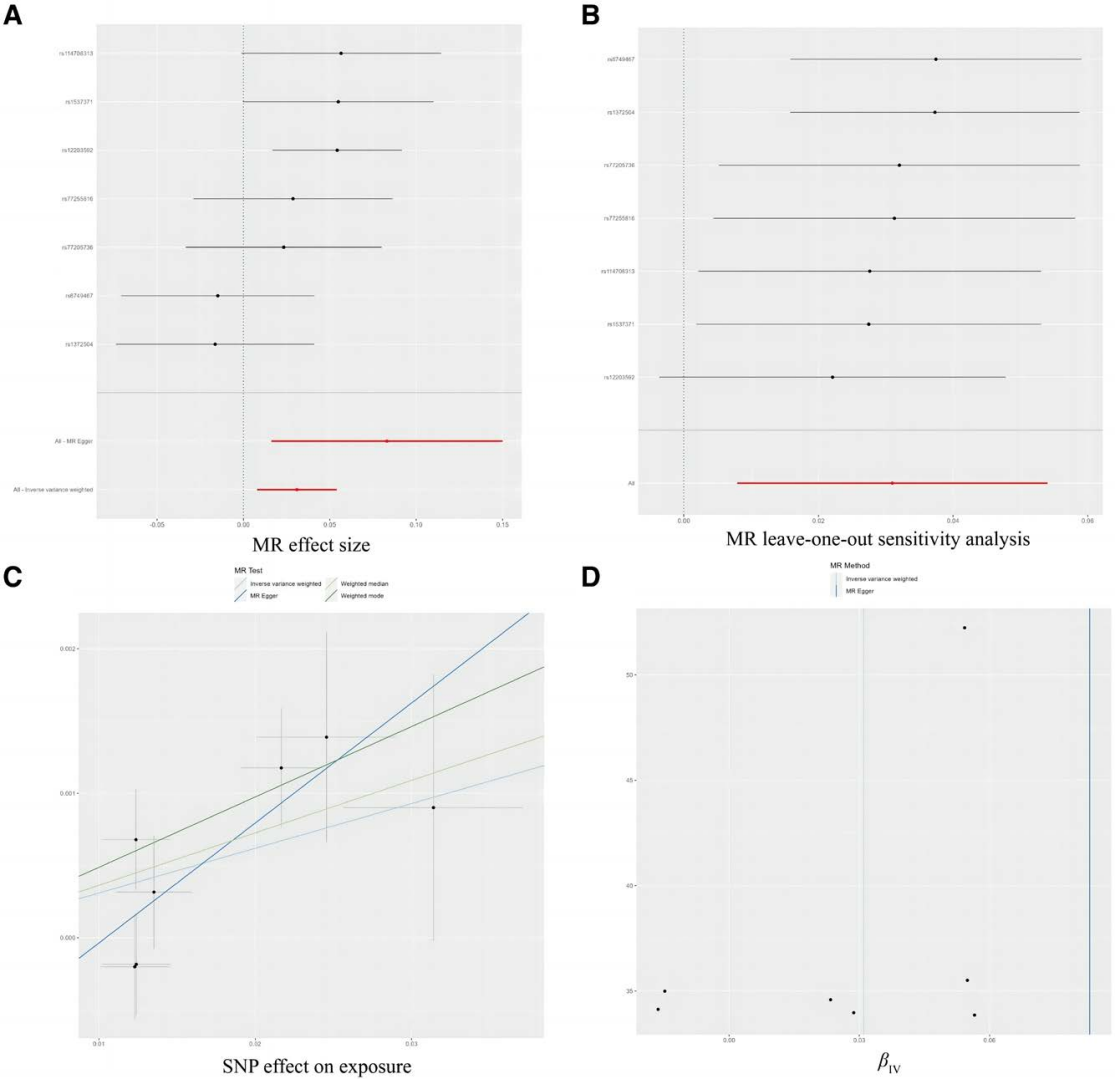
Forest plot (A), sensitivity analysis (B), scatter plot (C), and funnel plot (D) of the causal effect of PM2.5 on gingival pain risk. CP = chest pain, MR = Mendelian randomization, PM10 = particulate matter 10, SNPs = single nucleotide polymorphisms.

Regarding the relationship between air pollution and the risk of GP, the results of univariate MR analysis indicated that exposure to PM2.5 was causally associated with an increased risk of GP (OR_IVW_ = 1.031, 95% CI: 1.008–1.056, *P *= .008; OR_WM_ = 1.037, 95% CI: 1.009, 1.066, *P *= .010; Figs. 2 and 4). Although sensitivity analysis revealed an outlier SNP (rs12203592), the results of MR-PRESSO did not find an obvious influence of the outlier SNP on overall causal estimates (Table 2). Although the results from IVW method showed causal links of PM10 and PM2.5-10 with GP (PM10: OR_IVW_ = 1.015, 95% CI: 1.003–1.026, *P *= .012; PM2.5-10: OR_IVW_ = 1.013, 95% CI: 1.001–1.026, *P *= .034), but the WM method yielded the null hypothesis results (Fig. 2 and Figs. S4–S5, Supplemental Digital Content, https://links.lww.com/MD/P867). Furthermore, there was no relationships between exposure to NOx and the risk of GP (Fig. 2 and Fig. S6, Supplemental Digital Content, https://links.lww.com/MD/P867).

### 3.3. Direct causal effects of PM_2.5_ on CP and GP

To explore the causative impact of individual air contaminants on risk of CP and GP, MVMR analysis was carried out. After controlling for each PM, the findings indicated that the observed causal association between PM2.5 exposure and CP remained significant after correcting for the interactions of PM10 and PM2.5-10 (OR_IVW_ = 1.055, 95% CI: 1.018–1.094, *P *= .003). However, after correcting for PM10 and PM2.5-10, there was no indication of a direct relationship between PM2.5 and GP (OR_IVW_ = 1.024, 95% CI: 0.992–1.057, *P* = .138; Table S4, Supplemental Digital Content, https://links.lww.com/MD/P868).

Given the potential influences of behavioral factors (smoking and alcohol consumption) on disease risk of CP and GP, the additional MVMR analysis was conducted to infer the direct causality of PM2.5 exposure on CP and GP. The results demonstrated that there were still present causal associations of PM2.5 with the risk of CP (OR_IVW_ = 1.064, 95% CI: 1.031–1.100, *P* = 2 × 10^−04^) and GP (OR_IVW_ = 1.032, 95% CI: 1.004–1.059, *P* = .023), the specific results of the MVMR analysis were shown in Table S4, Supplemental Digital Content, https://links.lww.com/MD/P868.

## 4. Discussion

Air pollutants represent a pervasive environmental threat with heterogenous composition encompassing PM, gases, and organic compounds. While extensive epidemiological evidence has established their detrimental effects on cardiopulmonary health, the specific pathogenic mechanisms and site-specific pain manifestations remain underexplored.^[29]^ Conventional observational studies face challenges in disentangling confounding factors and reverse causality, particularly when assessing multifactorial conditions with shared risk profiles. Our study addresses these limitations through a two-step MR framework, providing the first genetic evidence for PM2.5 direct effects on CP and GP risk independent of particulate co-pollutants and behavioral confounders. Unlike previous observational studies, our study addresses 3 critical knowledge gaps in air pollution research. First, it elucidates the specific effects of PM exposure on multi-site pain conditions. Second, genetic instrumentation was employed to establish the temporal relationship between air pollution exposure and health outcomes. Finally, CP and GP were identified as novel pollution-sensitive endpoints.

The robustness of our findings is further substantiated by their persistence in multivariate models adjusting for PM10 and PM2.5-10, indicating that the unique physico-chemical properties of PM2.5, such as its smaller aerodynamic diameter, higher surface reactivity, and enhanced penetration, may contribute to site-specific pathological processes.^[30]^ Importantly, after controlling for smoking and alcohol consumption, this persistent association challenges the conventional paradigm of attributing the effects of air pollution exclusively to a common pathway associated with tobacco exposure.^[31]^ This distinction suggests that PM2.5 may operate through distinct biological mechanisms, necessitating long-term low-dose exposure rather than the acute pro-inflammatory effects observed with smoking.

CP is thought to be highly associated with cardiovascular system pathology, more than half of the CP patients were attributed to cardiovascular disease in the emergency department.^[32]^ A time-series study in China, demonstrated that high carbon monoxide (CO) and NOx exposures increased the risk of hospitalization in patients with coronary artery disease, but ozone exposure was negatively associated with the risk of hospitalization for coronary artery disease.^[33]^ Furthermore, evidence from a meta-analysis indicated that high levels of air pollutant exposure were positively associated with an elevated risk of cardiovascular disease, particularly in stroke and ischemic heart disease, and such harmful effects was much stronger in Asian countries and in specific populations (elderly and high body weight).^[34]^ In addition to the cardiovascular system, respiratory-related diseases are also associated with CP. A Canadian study demonstrated that high levels of NOx and CO exposure were associated with the number of asthma emergency visits, especially among children and elderly people over 75 years of age.^[35]^ Our results align with yet crucially extend prior reports linking PM2.5 to cardiovascular morbidity. While coronary calcification and ischemic events represent terminal outcomes, our identification of CP as a sentinel symptom suggests air pollution may induce subclinical inflammation manifesting as prodromal pain.^[32]^ This hypothesis was supported by experimental evidence of PM2.5-induced cytokine release (TNF-α, IL-6) and Th1 cell dysregulation, which confirms the biological validity of our findings.^[36,37]^

GP, often associated with gum pain in dental contexts, primarily stems from gum or periodontal lesions such as gingivitis or periodontitis, causing significant discomfort and distress. In recent years, emerging evidence suggests that air pollutants may contribute to the onset and progression of oral-related diseases. A cross-sectional study found that high levels of PM10 and ozone exposure increased the risk of periodontitis, whereas CO showed a negative association with periodontitis risk.^[38]^ Previous research has also linked PM2.5, NOx, and CO exposure to an increased risk of pulpitis visits.^[39]^ Furthermore, smoking is a well-established risk factor for periodontitis. A meta-analysis indicated that smokers are approximately 80% more likely to develop periodontitis compared to nonsmokers and quitters.^[40]^ Given the similarities between air pollutants and cigarette smoke, air pollutants may similarly promote GP, albeit potentially requiring longer exposure times compared to smoking. PM2.5 may alter oral microbial communities, predisposing to GP through dysregulated Th1/Th17 responses. The novel association with GP introduces an unexpected dimension to pollution’s health impacts. Unlike periodontitis’s established ties to local irritants, our demonstrationships.

Our study overcomes key limitations of prior pollution research. First, it employs an innovative approach to assess the causal connections between air pollutants and CP and GP from a genetic standpoint, thereby circumventing the pitfalls of reverse causality inherent in conventional observational research. Second, minimizes confounding through genetic instrumentation. Third, quantifies the effect of the independent of the behavior of variables. These analytical strengths are particularly salient in studying pain syndromes where subjective reporting biases frequently obscure exposure-outcome relationships.

## 5. Limitations and future direction

Despite these advancements, our research is not without limitations. A primary concern is the predominantly European ancestry of the datasets used in our MR analysis, which limits the applicability of our findings to other demographic groups with unique genetic profiles. Additionally, the chosen SNPs as IVs account for only a minor portion of the exposures examined, suggesting the presence of undiscovered or unreported SNPs that could affect causal estimations. Enhancing the sample size and integrating more rigorously validated IVs could significantly boost the statistical power and reliability of our causal deductions. In addition, the specific mechanisms through which air pollutants influence the onset and progression of these diseases may differ based on local environmental conditions, population genetics, and cultural practices, further research is needed to explore the transferability of these findings to other regions with similar or dissimilar geographical and climatic conditions.

Future investigations should focus to diversify the study population to include individuals from various ethnic backgrounds, thereby expanding the reach and depth of our observations. Longitudinal studies that monitor fluctuations in air pollutant concentrations and their subsequent impacts on health outcomes over extended periods are also crucial for gaining a more profound understanding of the temporal aspects of these associations.

## 6. Conclusions

Overall, our study establishes robust causal links between PM2.5 exposure and elevated risks of CP and GP. These findings underscore critical public health implications, advocating for enhanced air quality regulations and targeted preventive strategies to mitigate pollution-associated morbidity in susceptible populations.

## Author contributions

**Methodology:** Yao Li.

**Software:** Yin-Fei Luo.

**Validation:** Yin-Fei Luo, Yao Li, Yuan Liu, Xiao-Li Zhou.

**Visualization:** Xian-Pei Xiao, Rui Liang, Xiu-Ming Li, Yuan Liu, Xiao-Li Zhou.

**Writing – original draft:** Xian-Pei Xiao, Xiu-Ming Li.

**Writing – review & editing:** Zhi-Heng Li.

## Supplementary Material


