# Supplementary material for: Causal associations of air pollutants with chest and gingival pain: Genetic insight from Mendelian randomization study
Source: Medicine (Baltimore). 2025 Sep 5;104(36):e44258. doi: 10.1097/MD.0000000000044258 (PMC12419278; doi:10.1097/MD.0000000000044258)
Supplement: Supplementary file 1 [file medi-104-e44258-s001.docx]

| Table S1 Detailed information of datasets | | | | | |
| --- | --- | --- | --- | --- | --- |
| Data source | Phenotype | Sample size | Cases | Population | Adjustment |
| IEU Open GWAS project | Immune cells | - | - | European | - |
| IEU Open GWAS project（finn-b-CD2_MULTIPLE_MYELOMA_PLASMA_CELL_EXALLC） | Multiple myeloma and malignant plasma cell neoplasms (all cancers excluded) | 181354 | 598 | European | Males and Females |
| FinnGen（C3_MULT_MYELOMA_EXALLC） | Multiple myeloma (controls excluding all cancers) | 287714 | 585 | European | - |

| Table S2 Basic information on selected SNPs associated with multiple myeloma | | | | | | | | |
| --- | --- | --- | --- | --- | --- | --- | --- | --- |
|  | SNP | CHR | POS | EA/OA | EAF | beta | SE | *P* |
| 19901 | rs4291738 | NA | 40217816 | G/C | 0.0003 | 4.7731 | 2.5363 | 0.0598 |
| 29695 | rs77146037 | NA | 178226039 | G/A | 0.0005 | 3.2305 | 1.357 | 0.0173 |
| 3241 | rs114935210 | 5 | 134766276 | G/A | 0.0003 | 3.0266 | 2.031 | 0.1362 |
| 10836 | rs143928375 | NA | 88482297 | A/G | 0.0005 | 2.8506 | 1.3947 | 0.041 |
| 15230 | rs185778924 | NA | 149208450 | A/G | 0.0001 | 2.8445 | 1.2559 | 0.0235 |
| 15228 | rs185778924 | NA | 149208450 | A/G | 0.0001 | 2.8445 | 1.2559 | 0.0235 |
| 12393 | rs148727285 | NA | 49539940 | G/A | 0.0006 | 2.624 | 1.1697 | 0.0249 |
| 4500 | rs116661843 | NA | 178448859 | A/G | 0.0007 | 2.5966 | 1.2448 | 0.037 |
| 11606 | rs146445010 | 2 | 6597596 | C/G | 0.0008 | 2.5872 | 1.1367 | 0.0228 |
| 23717 | rs62447160 | NA | 36487489 | T/A | 0.0002 | 2.5647 | 2.8134 | 0.362 |
| 11 | ... | ... | ... | ... | ... | ... | ... | ... |
| 6082 | rs118006237 | NA | 100014674 | A/G | 0.0001 | -1.3925 | 2.978 | 0.6401 |
| 6081 | rs118006237 | NA | 100014674 | A/G | 0.0001 | -1.3925 | 2.978 | 0.6401 |
| 6080 | rs118006237 | NA | 100014674 | A/G | 0.0001 | -1.3925 | 2.978 | 0.6401 |
| 8709 | rs138425921 | 1 | 22721951 | T/C | 0.0001 | -1.4178 | 2.9092 | 0.626 |
| 26684 | rs73084801 | NA | 30080934 | T/C | 0.0023 | -1.6085 | 0.8912 | 0.0711 |
| 22459 | rs6001335 | NA | 38949961 | A/G | 0.0001 | -1.6374 | 3.1447 | 0.6026 |
| 12608 | rs149290264 | NA | 41618137 | T/C | 0.0087 | -1.7731 | 0.5132 | 0.0006 |
| 10093 | rs141950809 | NA | 92752895 | T/G | 0.0001 | -1.9337 | 4.9522 | 0.6962 |
| 5190 | rs117293367 | NA | 52043654 | T/G | 0.0002 | -3.2388 | 9.2157 | 0.7253 |
| 26835 | rs73168598 | NA | 33649515 | G/C | 0.0003 | -4.9174 | 3.8541 | 0.202 |

| Table S3 Basic information on selected SNPs associated with immune cells | | | | | | | | |
| --- | --- | --- | --- | --- | --- | --- | --- | --- |
|  | SNP | CHR | POS | EA/OA | EAF | beta | SE | *P* |
| 9033 | rs189058806 | 5 | 180091543 | T/A | 0.0044 | 32.88 | 20.12 | 0.1023 |
| 9107 | rs145294585 | 5 | 19919514 | C/T | 0.015 | 16.01 | 15.7 | 0.308 |
| 9028 | rs114121233 | 5 | 147197500 | C/T | 0.0129 | 15.93 | 11.5 | 0.166 |
| 9121 | rs148067960 | 6 | 129393491 | A/G | 0.0374 | 12.33 | 9.761 | 0.2068 |
| 9041 | rs76450314 | 17 | 71918099 | C/T | 0.0292 | 10.78 | 7.861 | 0.1702 |
| 9059 | rs114175375 | 3 | 52545924 | T/C | 0.0003 | 10.02 | 8.299 | 0.2275 |
| 9123 | rs191779135 | 4 | 82502357 | G/A | 0.0866 | 9.16 | 6.486 | 0.158 |
| 9111 | rs2578125 | 4 | 94881319 | T/C | 0.9616 | 8.901 | 9.158 | 0.3312 |
| 9130 | rs75295254 | 12 | 42337984 | C/T | 0.1389 | 8.023 | 5.274 | 0.1283 |
| 9112 | rs4877586 | 9 | 80938999 | A/G | 0.6521 | 7.017 | 3.818 | 0.0662 |
| 11 | ... | ... | ... | ... | ... | ... | ... | ... |
| 9129 | rs73218218 | 7 | 122188936 | A/G | 0.0263 | -11.59 | 11.14 | 0.2983 |
| 9013 | rs114175375 | 3 | 52545924 | T/C | 0.0003 | -14.24 | 73.06 | 0.8455 |
| 9105 | rs115253040 | 5 | 114515163 | T/C | 0.0329 | -21.71 | 10.3 | 0.0352 |
| 9127 | rs7170 | 1 | 36859163 | C/T | 0.0138 | -22.13 | 15.44 | 0.1518 |
| 9021 | rs7170 | 1 | 36859163 | C/T | 0.0138 | -25.62 | 11.45 | 0.0253 |
| 9132 | rs79361856 | 3 | 85173828 | G/T | 0.0148 | -25.87 | 15.15 | 0.0878 |
| 9016 | rs188873058 | 8 | 22025107 | G/C | 0.0048 | -26.3 | 18.23 | 0.1492 |
| 9037 | rs59341235 | 6 | 4964683 | C/T | 0.0007 | -47.6 | 45.54 | 0.296 |
| 9113 | rs59341235 | 6 | 4964683 | C/T | 0.0007 | -55.89 | 59.73 | 0.3495 |
| 9119 | rs114175375 | 3 | 52545924 | T/C | 0.0003 | -86.74 | 96.82 | 0.3704 |

| TableS 4 MR analysis of the causal relationship between immune cells and MM | | | | | | | | |
| --- | --- | --- | --- | --- | --- | --- | --- | --- |
| Exposure | Outcome | Classification | type | panel | Nsnp | Methods | OR(95%CI) | *P*-value |
| ebi-a-GCST90001448 | finn-b-CD2_MULTIPLE_MYELOMA_PLASMA_CELL_EXALLC | CD11c+ monocyte AC | Absolute count | cDC | 15 | Inverse variance weighted | 0.830(0.703-0.980) | 0.028046 |
| ebi-a-GCST90001516 | finn-b-CD2_MULTIPLE_MYELOMA_PLASMA_CELL_EXALLC | Im MDSC %CD33dim HLA DR- CD66b- | Relative count | Myeloid cell | 17 | Inverse variance weighted | 0.882(0.785-0.990) | 0.0336744 |
| ebi-a-GCST90001597 | finn-b-CD2_MULTIPLE_MYELOMA_PLASMA_CELL_EXALLC | CD8dim %T cell | Relative count | TBNK | 19 | Inverse variance weighted | 0.798(0.666-0.956) | 0.0141066 |
| ebi-a-GCST90001612 | finn-b-CD2_MULTIPLE_MYELOMA_PLASMA_CELL_EXALLC | CD8dim %leukocyte | Relative count | TBNK | 18 | Inverse variance weighted | 0.810(0.696-0.941) | 0.0060241 |
| ebi-a-GCST90001671 | finn-b-CD2_MULTIPLE_MYELOMA_PLASMA_CELL_EXALLC | CD39+ CD8br %CD8br | Relative count | Treg | 19 | Inverse variance weighted | 1.152(1.017-1.305) | 0.0257477 |
| ebi-a-GCST90001744 | finn-b-CD2_MULTIPLE_MYELOMA_PLASMA_CELL_EXALLC | CD20 on CD20- CD38- | MFI | B cell | 15 | Inverse variance weighted | 1.281(1.050-1.563) | 0.0145223 |
| ebi-a-GCST90001764 | finn-b-CD2_MULTIPLE_MYELOMA_PLASMA_CELL_EXALLC | CD24 on CD24+ CD27+ | MFI | B cell | 3 | Inverse variance weighted | 0.705(0.508-0.977) | 0.0355335 |
| ebi-a-GCST90001819 | finn-b-CD2_MULTIPLE_MYELOMA_PLASMA_CELL_EXALLC | CD38 on transitional | MFI | B cell | 22 | Inverse variance weighted | 1.192(1.040-1.365) | 0.0114067 |
| ebi-a-GCST90002024 | finn-b-CD2_MULTIPLE_MYELOMA_PLASMA_CELL_EXALLC | CD4 on naive CD4+ | MFI | Maturation stages of T cell | 20 | Inverse variance weighted | 0.827(0.700-0.976) | 0.024739 |
| ebi-a-GCST90002094 | finn-b-CD2_MULTIPLE_MYELOMA_PLASMA_CELL_EXALLC | CD11b on Mo MDSC | MFI | Myeloid cell | 16 | Inverse variance weighted | 0.892(0.803-0.990) | 0.0316926 |
| ebi-a-GCST90002111 | finn-b-CD2_MULTIPLE_MYELOMA_PLASMA_CELL_EXALLC | HLA DR on CD33dim HLA DR+ CD11b- | MFI | Myeloid cell | 18 | Inverse variance weighted | 0.859(0.759-0.973) | 0.0165014 |
| ebi-a-GCST90001448 | C3_MULT_MYELOMA_EXALLC | CD11c+ monocyte AC | Absolute count | cDC | 15 | Inverse variance weighted | 0.829(0.700-0.981) | 0.0289816 |
| ebi-a-GCST90001516 | C3_MULT_MYELOMA_EXALLC | Im MDSC %CD33dim HLA DR- CD66b- | Relative count | Myeloid cell | 17 | Inverse variance weighted | 0.870(0.768-0.987) | 0.0304294 |
| ebi-a-GCST90001597 | C3_MULT_MYELOMA_EXALLC | CD8dim %T cell | Relative count | TBNK | 19 | Inverse variance weighted | 0.800(0.659-0.972) | 0.0248802 |
| ebi-a-GCST90001612 | C3_MULT_MYELOMA_EXALLC | CD8dim %leukocyte | Relative count | TBNK | 18 | Inverse variance weighted | 0.780(0.671-0.907) | 0.0012758 |
| ebi-a-GCST90001671 | C3_MULT_MYELOMA_EXALLC | CD39+ CD8br %CD8br | Relative count | Treg | 20 | Inverse variance weighted | 1.207(1.073-1.356) | 0.0016649 |
| ebi-a-GCST90001744 | C3_MULT_MYELOMA_EXALLC | CD20 on CD20- CD38- | MFI | B cell | 15 | Inverse variance weighted | 1.212(1.006-1.459) | 0.0426247 |
| ebi-a-GCST90001764 | C3_MULT_MYELOMA_EXALLC | CD24 on CD24+ CD27+ | MFI | B cell | 3 | Inverse variance weighted | 0.704(0.506-0.981) | 0.0380622 |
| ebi-a-GCST90001819 | C3_MULT_MYELOMA_EXALLC | CD38 on transitional | MFI | B cell | 22 | Inverse variance weighted | 1.158(1.014-1.323) | 0.0304985 |
| ebi-a-GCST90002024 | C3_MULT_MYELOMA_EXALLC | CD4 on naive CD4+ | MFI | Maturation stages of T cell | 20 | Inverse variance weighted | 0.770(0.653-0.909) | 0.0020203 |
| ebi-a-GCST90002094 | C3_MULT_MYELOMA_EXALLC | CD11b on Mo MDSC | MFI | Myeloid cell | 16 | Inverse variance weighted | 0.887(0.799-0.984) | 0.0231166 |
| ebi-a-GCST90002111 | C3_MULT_MYELOMA_EXALLC | HLA DR on CD33dim HLA DR+ CD11b- | MFI | Myeloid cell | 18 | Inverse variance weighted | 0.875(0.784-0.977) | 0.0175636 |

| Table S5 MR analysis of the causal relationship between MM and immune cells | | | | | | | | |
| --- | --- | --- | --- | --- | --- | --- | --- | --- |
| Exposure | Outcome | Classification | type | panel | Nsnp | Methods | OR(95%CI) | *P*-value |
| finn-b-CD2_MULTIPLE_MYELOMA_PLASMA_CELL_EXALLC | ebi-a-GCST90001723 | CD19 on CD24+ CD27+ B cell | MFI | B cell | 16 | Inverse variance weighted | 1.035(1.001-1.069) | 0.0426827 |
| finn-b-CD2_MULTIPLE_MYELOMA_PLASMA_CELL_EXALLC | ebi-a-GCST90002005 | CD16 on CD14+ CD16+ monocyte | MFI | Monocyte | 16 | Inverse variance weighted | 1.048(1.014-1.084) | 0.00589 |
| C3_MULT_MYELOMA_EXALLC | ebi-a-GCST90001723 | CD19 on CD24+ CD27+ B cell | MFI | B cell | 14 | Inverse variance weighted | 1.045(1.002-1.090) | 0.0410265 |
| C3_MULT_MYELOMA_EXALLC | ebi-a-GCST90002005 | CD16 on CD14+ CD16+ monocyte | MFI | Monocyte | 14 | Inverse variance weighted | 1.039(1.002-1.078) | 0.0371445 |
