# Supplementary material for: Causal associations of air pollutants with chest and gingival pain: Genetic insight from Mendelian randomization study
Source: Medicine (Baltimore). 2025 Sep 5;104(36):e44258. doi: 10.1097/MD.0000000000044258 (PMC12419278; doi:10.1097/MD.0000000000044258)

**Supplementary Fig. 1** Scatter plots of immune cells and MM risk (IEU dataset)


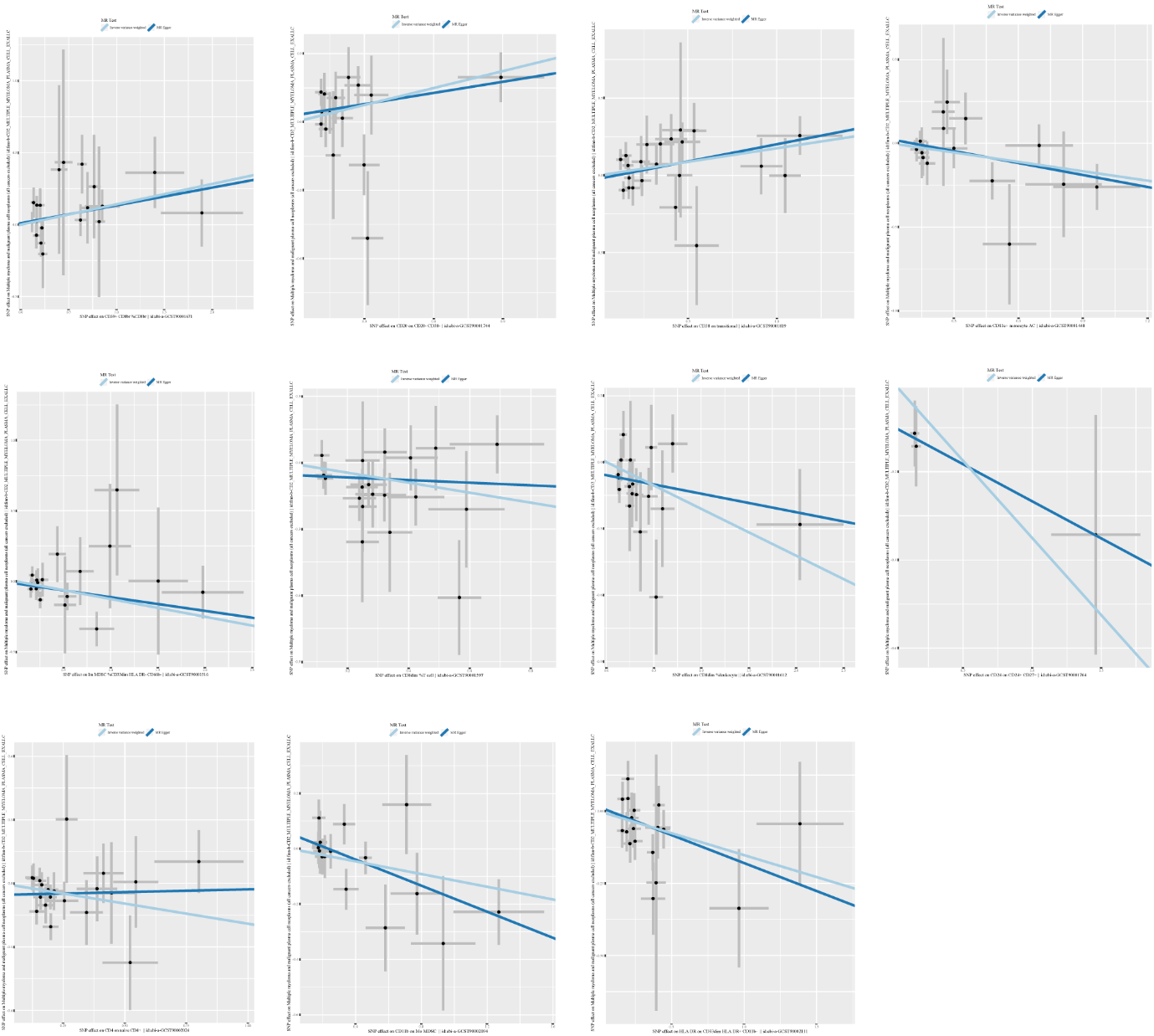


**Supplementary Fig. 2** Funnel plots of immune cells and MM risk (IEU dataset)


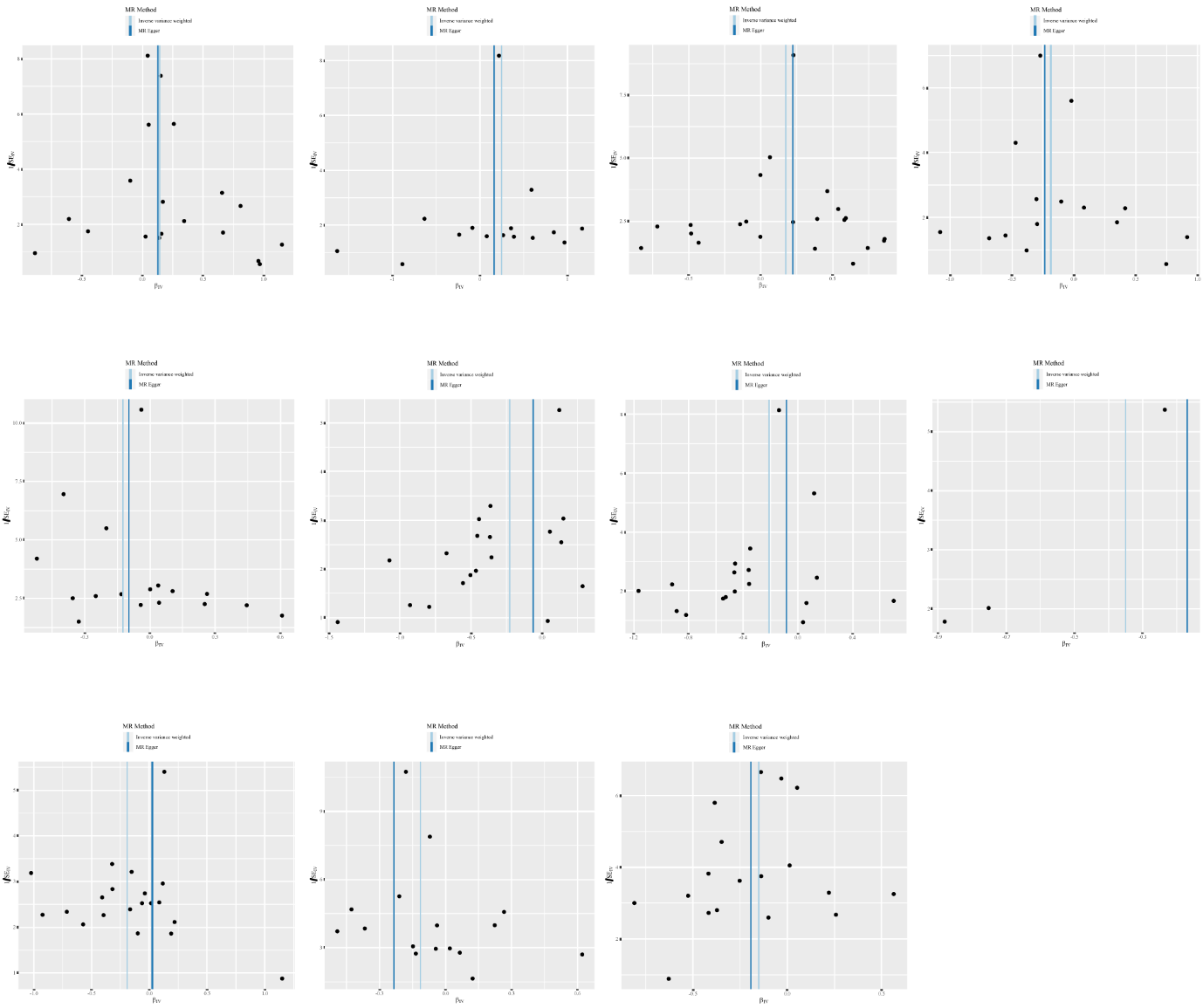


**Supplementary Fig. 3** Scatter plots of immune cells and MM risk (FinnGen dataset)


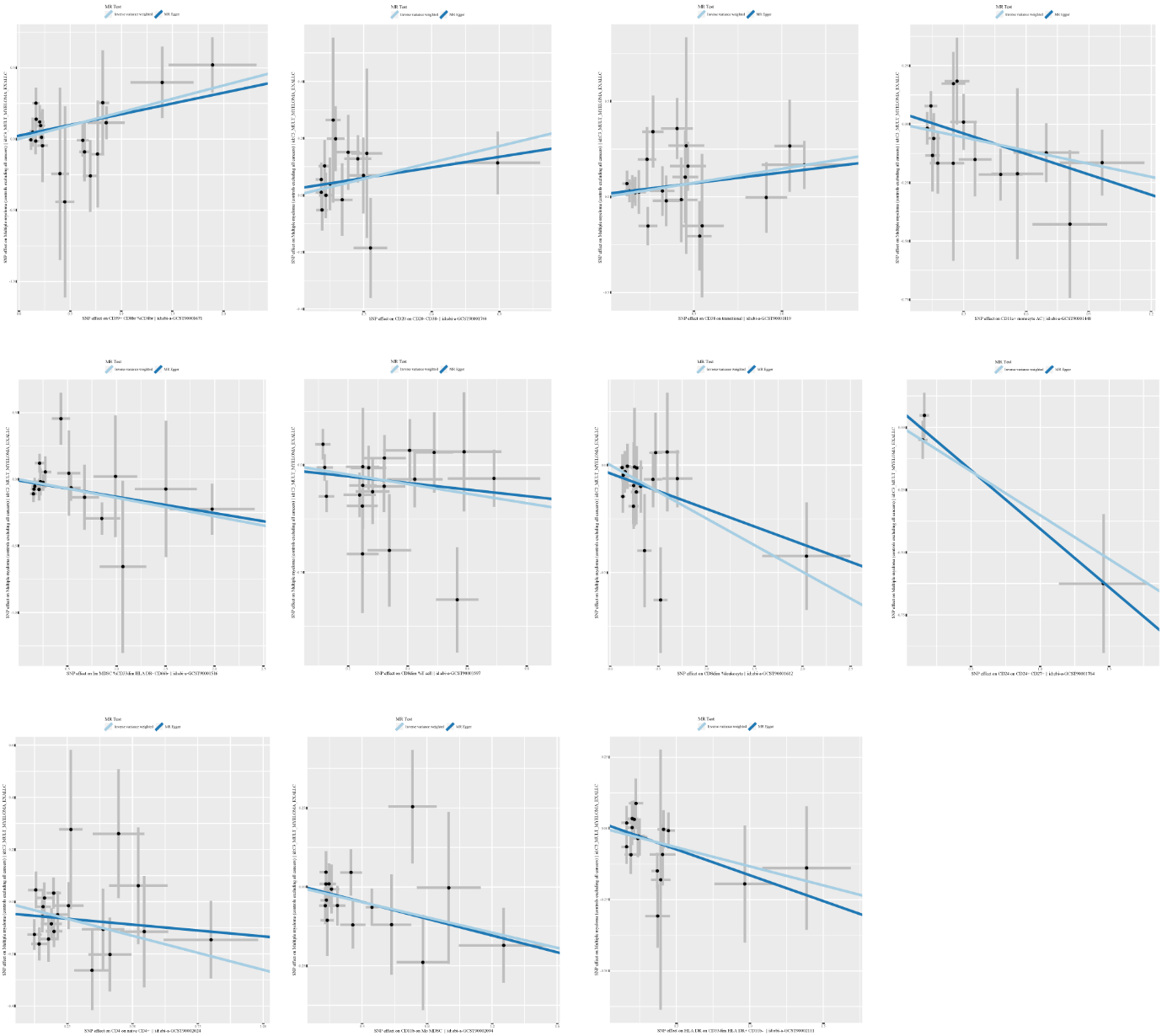


**Supplementary Fig. 4** Funnel plots of immune cells and MM risk (FinnGen dataset)


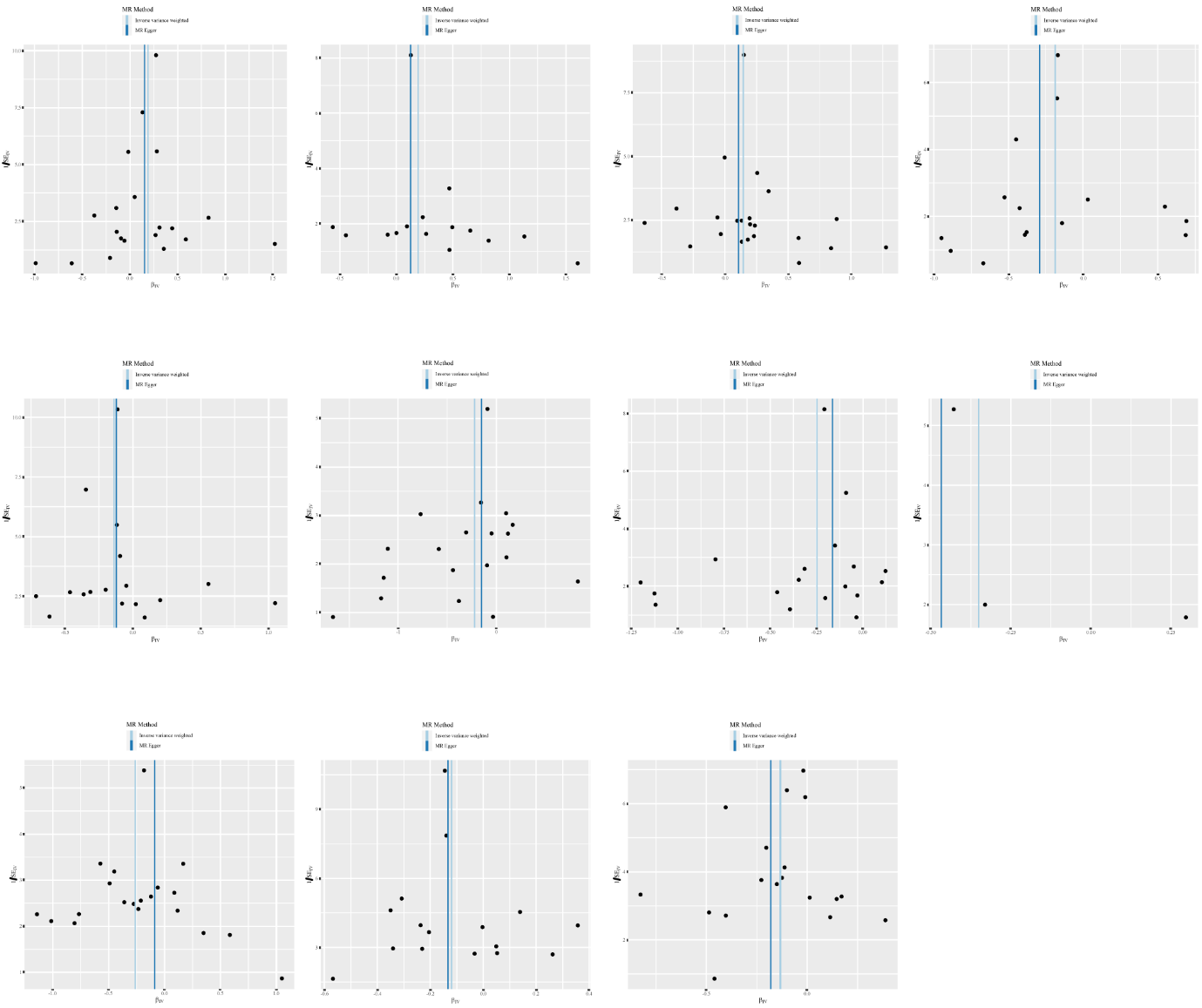


**Supplementary Fig. 5** Leave-one-out plots of immune cells and MM risk (IEU dataset)


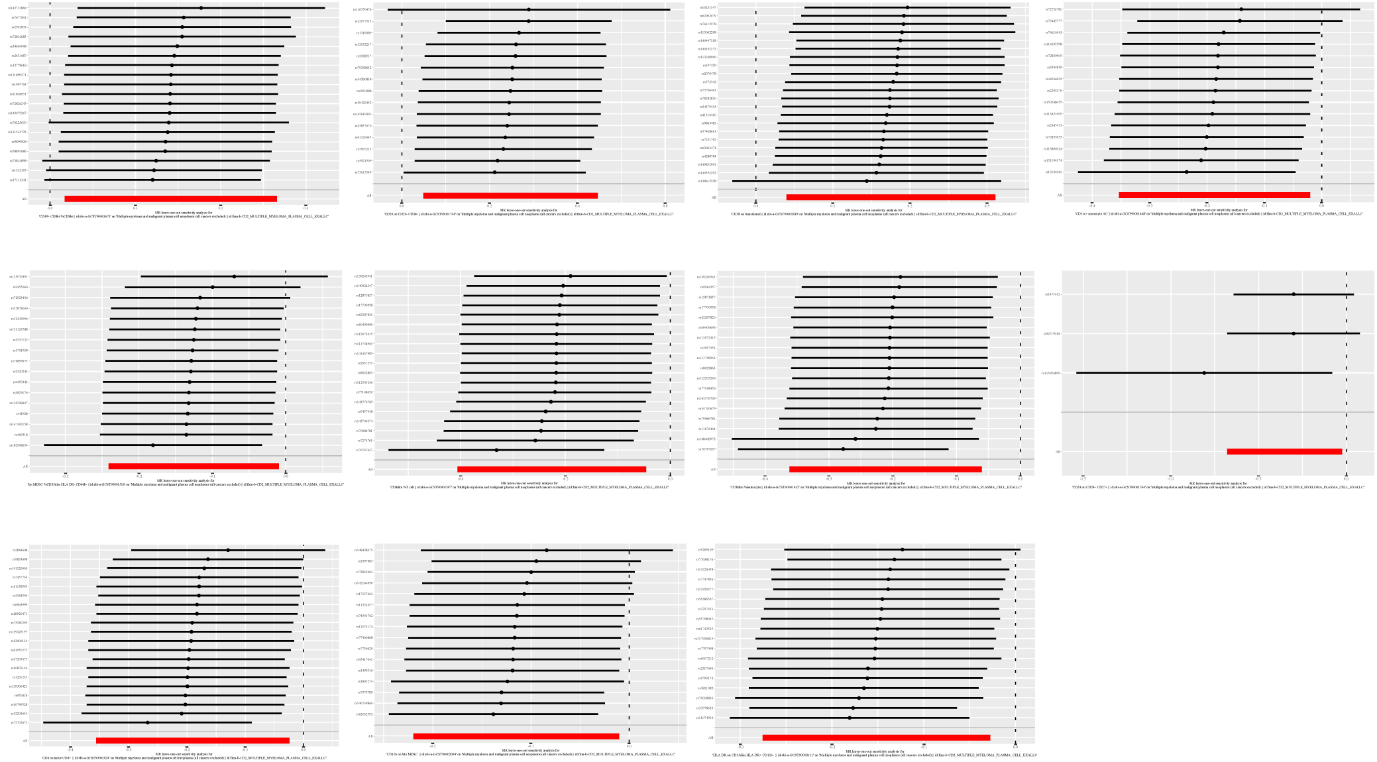


**Supplementary Fig. 6** Leave-one-out plots of immune cells and MM risk (FinnGen dataset)


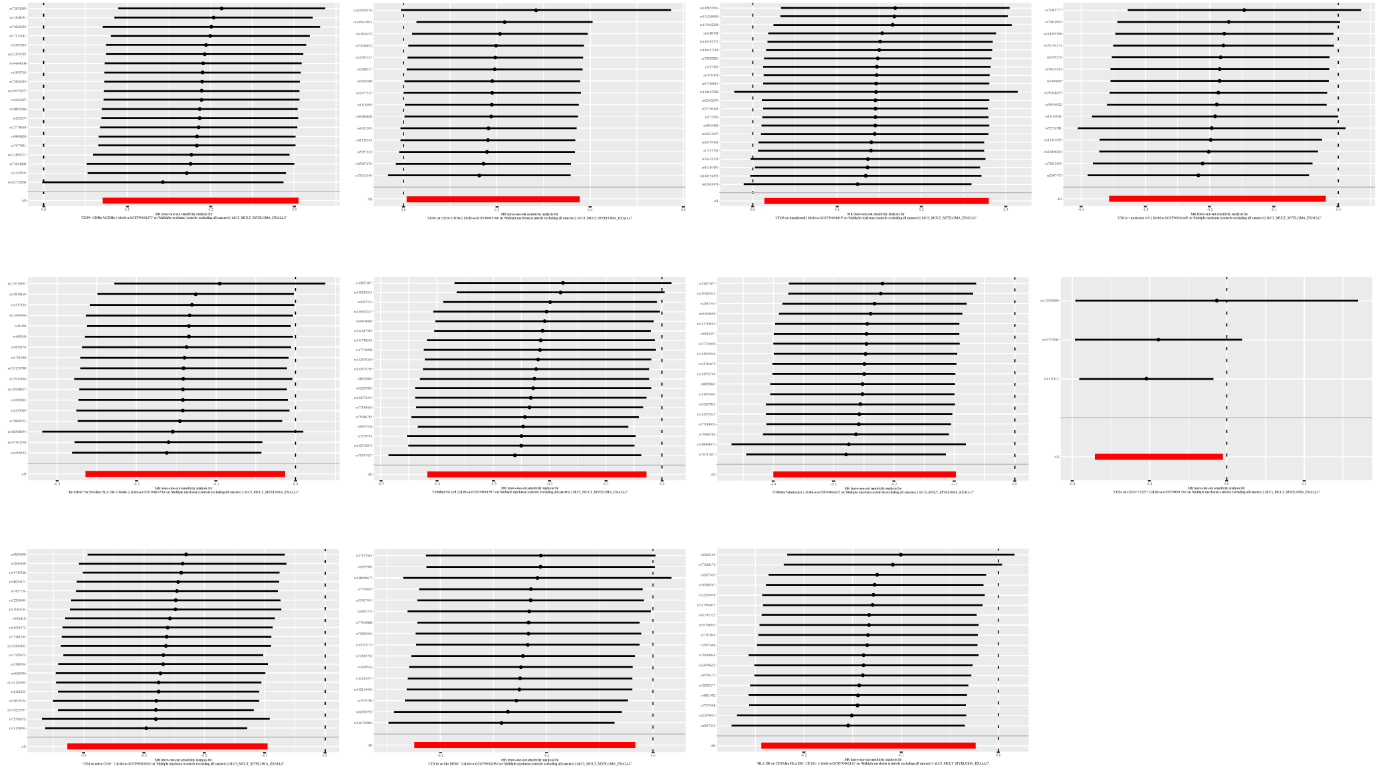


**Supplementary Fig. 7** Scatter and funnel plots of MM onset against immunophenotypes


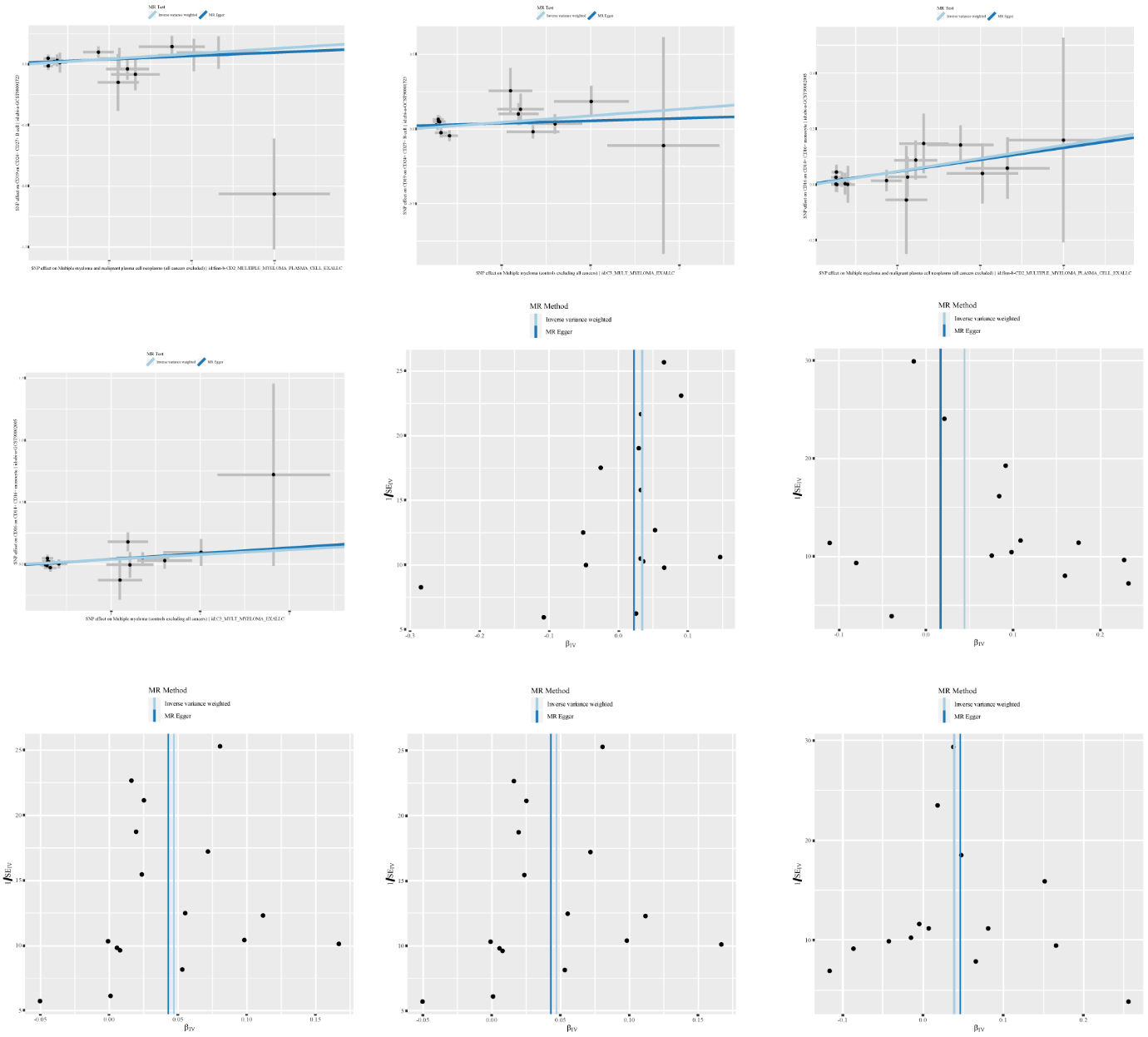

Supplement: Supplementary file 2 [file medi-104-e44258-s002.docx]
